# DIAMIN: a software library for the distributed analysis of large-scale molecular interaction networks

**DOI:** 10.1186/s12859-022-05026-w

**Published:** 2022-11-11

**Authors:** Lorenzo Di Rocco, Umberto Ferraro Petrillo, Simona E. Rombo

**Affiliations:** 1 Department of Statistics, University of Rome La Sapienza, Rome, Italy; 2 Department of Mathematics and Computer Science, University of Palermo, Palermo, Italy

**Keywords:** Biological networks, Big data analytics, Molecular interactions, Large scale networks

## Abstract

**Background:**

Huge amounts of molecular interaction data are continuously produced and stored in public databases. Although many bioinformatics tools have been proposed in the literature for their analysis, based on their modeling through different types of biological networks, several problems still remain unsolved when the problem turns on a large scale.

**Results:**

We propose DIAMIN, that is, a high-level software library to facilitate the development of applications for the efficient analysis of large-scale molecular interaction networks. DIAMIN relies on distributed computing, and it is implemented in Java upon the framework Apache Spark. It delivers a set of functionalities implementing different tasks on an abstract representation of very large graphs, providing a built-in support for methods and algorithms commonly used to analyze these networks. DIAMIN has been tested on data retrieved from two of the most used molecular interactions databases, resulting to be highly efficient and scalable. As shown by different provided examples, DIAMIN can be exploited by users without any distributed programming experience, in order to perform various types of data analysis, and to implement new algorithms based on its primitives.

**Conclusions:**

The proposed DIAMIN has been proved to be successful in allowing users to solve specific biological problems that can be modeled relying on biological networks, by using its functionalities. The software is freely available and this will hopefully allow its rapid diffusion through the scientific community, to solve both specific data analysis and more complex tasks.

## Background

The intensive interplay of molecular components is at the basis of cellular life. Understanding how genes, proteins and RNAs interact each other allows to uncover complex mechanisms which underlie the occurrence and progress of complex diseases [1–3]. With the advent of high throughput and computational techniques, massive amounts of interaction data have been produced and collected in publicly available databases (e.g., Intact [4], String [5]). Molecular interaction data are usually modelled by *molecular interaction networks* (MIN, for short), such that nodes are associated with cellular components, and edges represent their pairwise interactions, experimentally discovered in laboratory or predicted computationally [6].

Different types of molecular interaction networks (e.g., protein–protein interaction networks) have been broadly studied in the literature, showing to be successful also in real-life applications [7]. Moreover, a number of software tools have been proposed and are commonly used for their analysis and visualization, the most used of which is Cytoscape [8]. However, interaction data collected in public databases have impressively increased in the last few years, bringing such networks to reach huge sizes, with thousands proteins and millions interactions to be processed. Unfortunately, this has started to make the analysis of molecular interaction networks yet more challenging, and often out-of-reach for standard computing platforms.

A possible solution is to carry out the analysis of these very large networks by identifying independent tasks to be solved, in parallel, across the nodes of a distributed computing system. However, further significant issues would need to be solved in this case. Among them, there is the definition of a strategy for partitioning in a balanced way the elements of the interaction network under analysis on the nodes of the distributed system. Then, the development of distributed algorithms able to carry out the target analysis in parallel over these nodes, thus that each node may work mostly on its local data, while keeping small the amount of data to be exchanged with the other nodes.

Indeed, these issues are not easy to tackle as they require a profound knowledge of the way a distributed system works, as well as non-trivial programming skills. For these reasons, the usage of distributed computing in the analysis of molecular interaction networks has been pretty limited so far, despite its potential and possible social impact have been widely pointed out in the literature (see, e.g., [9]).

To address the above problems, we propose DIAMIN, a high-level software library implemented in Java, based on the Object-Oriented Programming paradigm and built on the Apache Spark framework. The main goal of this library is to enable efficient distributed analysis of large molecular interaction networks, both for users with programming skills (PSU) and for data analysts (DAU). In both cases, the user does not need any special knowledge of distributed computing to fully utilise the features provided by the library. Instead, it is the library provided that takes care of the various problems that arise when working in a distributed environment, such as balancing the distribution of the elements of an input interaction network among the nodes of a distributed system and processing them in parallel.

In summary, DIAMIN utilities allow the user to perform the following three main classes of actions.

(PSU, DAU) Build and query molecular interaction networks, with nodes representing cellular components and edges their interactions, starting from the most common file formats available in public databases that store annotations on molecular interactions (e.g., *mitab* files).
(DAU) Perform basic analysis of molecular interaction networks, through a set of useful functionalities (e.g., neighborhood extraction).
(PSU) Compose and modify in a simple way the suitable primitives provided by the platform, to perform more complex analysis and/or to solve specific problems (e.g., network alignment).

It is worth pointing out that many of such actions cannot be accomplished by the state of the art currently available tools (e.g., Cytoscape [8]), especially when large-scale networks (order of million edges) have to be analyzed in their entirety. Therefore, to the best of our knowledge, DIAMIN is the first and only software library specifically designed to allow MIN analysis in the distributed.

The remaining part of this section is devoted to provide some basic definitions on networks and graphs, as well as the main notions on big data frameworks and methodologies the proposed library relies on.

### Molecular interaction networks and basics on graphs

As defined in several papers of the literature (see, e.g., [6, 10, 11]) a Molecular Interaction Network (MIN) ${{\mathcal {N}}}=\langle V, E \rangle$ is an undirected graph such that *V* is the set of nodes, representing cellular components (e.g., proteins, mRNA, genes, etc.), and *E* is the set of edges. An edge occurs between two nodes if a physical interaction between the two corresponding cellular components has been found in laboratory or computationally predicted, as stored in annotated interaction databases. If each edge of a MIN has a label (usually, a real number), the MIN is *labelled*. We recall that, given the edge $(v_i, v_j) \in E$, it is *incident* to both nodes $v_i$ and $v_j$. Also, $v_i$ and $v_j$ are *adjacent*. Two edges are adjacent as well if they share a node. The *degree* of a node *v* is the number of edges incident to *v*.

The following definitions hold.

#### Definition 1

x-Path. *Given a MIN *${{\mathcal {N}}}=\langle V, E \rangle$,*an* *x*-*Path is a sequence* $(e_1, e_2, \ldots , e_h)$ *of* *h* *edges such that*:

- $e_i$ *and* $e_{i+1}$ *are adjacent, for each* $i=1, \ldots , h-1$;
- *the product of labels of the edges* $(e_1, e_2, \ldots , e_h)$ *is greater than* *x*, *if* ${{\mathcal {N}}}$ *is labelled*.
- $h=x$, *if *${{\mathcal {N}}}$ *is not labelled*.

#### Definition 2

Density. *The density of a MIN *${{\mathcal {N}}}=\langle V, E \rangle$ *is defined as*:

$$\begin{aligned} \frac{2|E|}{|V|(|V|-1)} \end{aligned}$$

*where* |*E*| *and* |*V*| *are the number of edges and nodes, respectively*.

#### Definition 3

Closeness. *Given a MIN* ${{\mathcal {N}}}=\langle V, E \rangle$ *and an interactor* $p \in V$, *the closeness of p is defined as*:

$$\begin{aligned} \sum _{y\in V\setminus \{p\}} \frac{1}{d(y,p)} \end{aligned}$$

*where* *d*(*y*, *p*) *is the length of the shortest path between* *y* *and* *p*.

Given a node *p* of a MIN ${{\mathcal {N}}}$, it can be useful in many applications to consider nodes at a certain distance from *p* on ${{\mathcal {N}}}$. In particular, this may be important for both unlabelled MIN, and for labelled ones. In the latter case, we assume that labels are real values in the range [0, 1]. Then, one can refer to the previous definitions, and consider the *x*-*Neighborhood* of *p* as the set of all nodes $\{v_1, v_2, \ldots , v_h\}$ in $\mathcal{N}$ for which there exists a *y*-Path between $v_i$ ($i=1, \ldots , h$) and *p*, such that $y \ge x$.

#### Definition 4

Closest Component. *Given a MIN* ${{\mathcal {N}}}=\langle V, E \rangle$ *and a partition* ${{\mathcal {A}}}$ *of* *N*, *the closest component to a subset of interactors* $S \subset N$ *is the set* ${{\mathcal {C}} \in {{\mathcal {A}}}}$ *with the largest number of interactors in common with* *S*:

$$\begin{aligned} {{\mathcal {C}}}=\arg \max _{A \in {{\mathcal {A}}}} |A\cap S | \end{aligned}$$

### Big data technologies

#### Distributed computing

Distributed Computing is an environment in which a group of independent, heterogeneous and geographically dispersed computer systems take part to solve a complex problem, each by solving a part of solution and then combining the result from all computers. These systems are loosely coupled systems coordinately working for a common goal [12]. The use of distributed computing is particularly helpful when working with big data, as it allows both to manage very large amounts of data by partitioning them among the nodes of a distributed system, and to significantly speed up their analysis thanks to parallel computation.

#### The MapReduce paradigm

*MapReduce* [13] is a paradigm for the processing of large amounts of data on a distributed computing infrastructure. Assuming that the input data is organized as a set of $\langle$ ${key}, {value}$ $\rangle$ pairs, the paradigm is based on the definition of two functions. The *map* function, which processes an input $\langle$ ${key}, {value}$ $\rangle$ pair and returns a (possibly empty) intermediate set of $\langle$ ${key}, {value}$ $\rangle$ pairs. The *reduce* function, which merges all the intermediate values sharing the same key to form a (possibly smaller) set of values. These functions are run, as tasks, on the nodes of a distributed computing framework. A complex algorithm can be implemented by running a sequence containing an arbitrary number of map and/or reduce functions. The management of map and reduce functions/results are transparently handled by the underlying framework (*implicit parallelism*), with no burden on the programmer.

#### Apache spark

Apache Spark [14] is one of the most popular engines for large scale data processing, based on RDDs (Resilient Distributed Datasets) and DataFrames. These are distributed memory abstractions that let programmers perform in-memory computations on large clusters in a fault-tolerant manner. The physical architecture of a Spark Cluster is characterized by a master node that supervises a series of workers nodes through daemon processes. Data are usually partitioned among the worker nodes and MapReduce is also supported.

#### The GraphX and GraphFrame libraries

Apache Spark includes two high-level APIs useful for writing applications able to process large-scale graphs, distributed across a computer cluster: GraphX [15] and GraphFrame [16]. These two APIs mainly differ in the data structures used to represent an input graph.

GraphX extends Spark’s RDDs to introduce a new graph abstraction, called Resilient Distributed Graph. On top of this data structure, a set of operations is provided to simplify distributed graph construction and processing. It models the edges and the vertices of the graph using two different RDDs: VertexDataTable and EdgeTable. The former stores, for each vertex of the graph, a unique vertex id and a set of user-defined data. The latter stores, for each edge of the graph, the source vertex id, the destination vertex id, a set of user-defined data and a virtual partition identifier (pid). The elements of EdgeTable instances are partitioned across the nodes of a Spark Cluster according to their pid attribute, while the elements of VertexDataTable instances are partitioned according to their vertex id.

GraphX generates a further RDD, called VertexMap, that provides a mapping from the id of a vertex to the partitions that contain adjacent edges. Since graph algorithms often require to explore the neighborhood of each vertex, an efficient strategy aims to minimize communications between different graph partitions, ensuring at the same time a balanced computation workload.

GraphFrame unifies graph analytics and relational queries using a graph representation based on Spark DataFrames. These data structures model a dataset as a distributed relational table, to be queried using an SQL-like language.

GraphFrame represents a distributed graph using two DataFrames: a DataFrame holding all vertices and a DataFrame holding all edges. The main difference with respect to GraphX is the availability of a graph search operator that accepts, as input, a pattern specifying the structure of the subgraph being searched and returns a new DataFrame containing all edges and/or vertices of the original graph matching the provided pattern.

*PREGEL.* PREGEL [17] is a node-centric programming model where the developer can implement an algorithm from the perspective of a node, rather than of the whole graph. Namely, it allows to code the behavior of each node of a graph, provided that it can exchange messages with the other nodes it is connected to. Execution takes place in several rounds and stops when every computation at each node votes to halt.

*Pregel* is based on a multi-iteration procedure where adjacent nodes communicate by means of messages. Within each iteration (called superstep) a set of active nodes update their attributes according to a user-defined function which elaborates the message sent by the adjacent nodes. The sequence of supersteps continue until no more active nodes are detected (i.e there are no messages to forward to the next iteration). The pregel schema is meant to be extremely suitable for per-node computations over many machines.

The GraphX API provides the tools to implement a Pregel through parallel computations on the triplets of the graph. A triplet is made of an edge, its source and its destination node. The high level of abstraction requires the user to specify three custom functions:

- Vertex-Programme function (Vprog);
- Merge-Message function (MergeMsg);
- Send-Message function (SendMsg).

*MergeMsg* aggregates for each node the messages delivered by the adjacent nodes. The output is processed by the *Vprog* function to update the node data. At the end, *SendMsg* generates the message to be forwarded to the next superstep, selecting those nodes that will be active. Moreover, the GraphX API allows to fix other parameters, such as the maximum number of iterations and the direction of messages propagation.

*Neo4J.* Neo4J is a NoSQL database designed for efficiently managing and querying highly connected data, by means of a graph-based representation. It can be deployed as a distributed database, using sharding, when it is needed to analyze huge networks. Cypher is the language included in Neo4J and designed for querying stored data and for calling the built-in functions implementing many useful graph algorithms. Neo4J includes also software components for a deep integration with Apache Spark, that allows users to easily implement distributed software pipelines using Neo4J as a data storage.

## Implementation

The library has been implemented as a collection of Java classes. This language has been chosen against the other two languages supported by Apache Spark, Scala and Python, due to the fact that it provides an optimal trade-off between the need for a robust Object-Oriented implementation (unlike Python) and the use of a widely known programming language (unlike Scala).

**Fig. 1:**
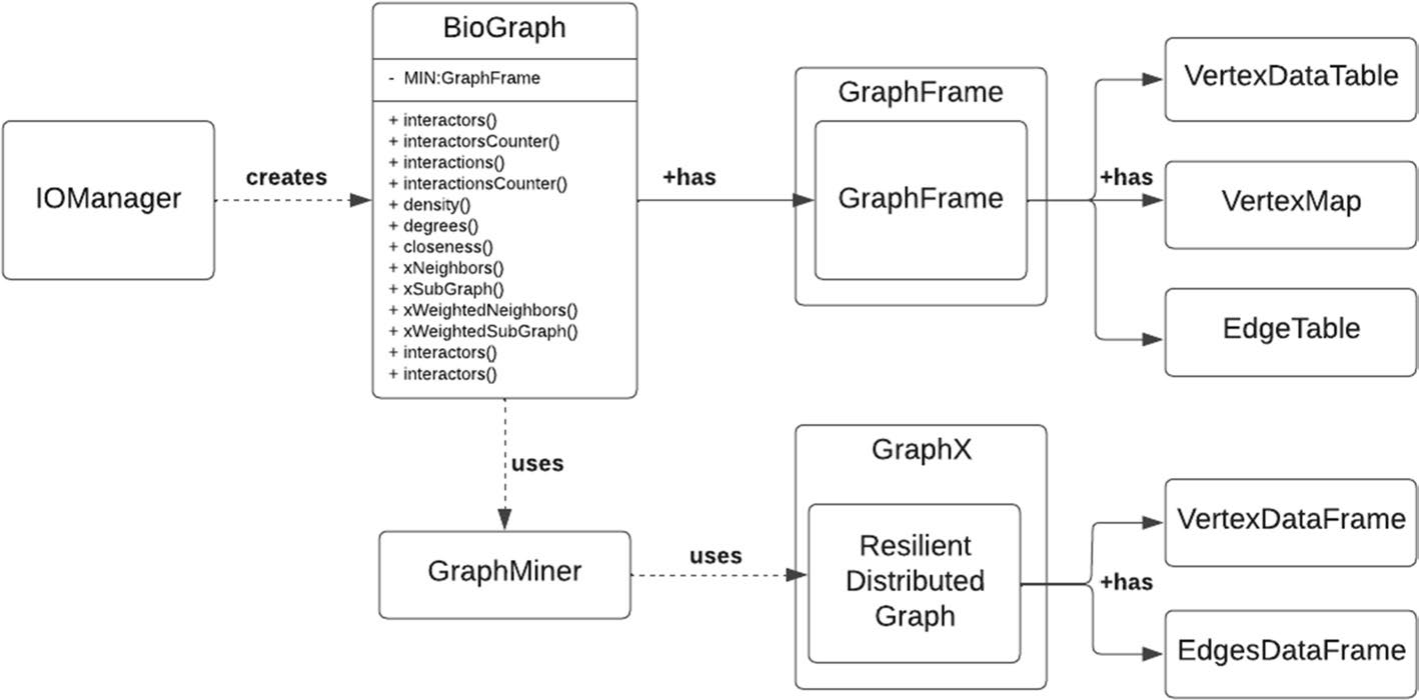
High-level design of the DIAMIN architecture, depicted as a class diagram. It reports the main classes used by the library and their interactions

We describe in the following the main two classes it contains: BioGraph and IOmanager. A class diagram reporting a high-level description of the architecture of MIN is available in Fig. 1. The library also includes other classes to manage import/export routines or to represent the basic concepts of a graph structure.

BioGraph. The core component of MIN is the BioGraph class. It enables the management and processing of a MIN using a distributed representation. From the outside, an instance of this class appears to enclose a MIN and provides a set of methods and algorithms for interacting with it. From the inside, the enclosed MIN is not local to the instance of the class, but it is instead implemented by two different distributed Spark data structures, each containing nodes and edges of the MIN. Thus, the BioGraph instance contains only one stub pointing to the various partitions of MIN. As a consequence, the distributed nature of this data structure is completely invisible to the end user.

Similarly, methods supported by this class are also represented as standard methods, but they are implemented as distributed algorithms. This allows the user to take advantage of distributed computing for processing very large MIN networks without having any knowledge of distributed programming.

In details, BioGraph supports three main types of operations:

- *MIN statistics* useful to summarize the properties of a whole MIN network or of some of its parts by means of centrality measures;
- *MIN analysis* useful to explore the interaction, in terms of connectivity and weighted connectivity, between different parts of a same MIN network. Includes path-finding and connected components algorithms;
- *MIN exploration* useful to explore the structure a MIN network by traversing it or by giving access to some of its parts, even according to user-defined conditions;

Functions belonging to the first type cover tasks typical for the analysis of any type of graphs, while the others are designed to implement higher-level algorithms, conceived for the solution of specific domain-driven problems. The considered domain-driven problems focus mainly on cellular components connectivity and the evaluation of the related connected components.

In the following, a list of the main algorithms available within this class is provided, where each algorithm is implemented as a method. It is worth pointing out that all these methods, when run, implicitly use the MIN stored in the class itself as input.

- **interactors()**
  - *Input: * -
  - *Description: * writes on an external text file the unique id or the name of all the interactors (i.e., the nodes) of the underlying graph.
- **interactorsCounter()**
  - *Input: -*
  - *Description:* returns the number of interactors of the underlying graph.
- **interactions()**
  - *Input: -*
  - *Description:* writes on an external text file the unique id or the name of all interactions (i.e., edges) of the underlying graph.
- **interactionsCounter()**
  - *Input: -*
  - *Description:* returns the number of interactions (i.e., the edges) of the underlying graph.
- **density()**
  - *Input: -*
  - *Description:* returns the density index of the underlying graph.
- **degrees()**
  - *Input: -*
  - *Description:* returns the list of interactors of the underlying graph, with their associated degree.
- **closeness()**
  - *Input:* an interactor *i* of the underlying graph.
  - *Description:* returns the closeness of the interactor *i*.
- **xNeighbors()**
  - *Input:* a subset *S* of interactors of the (unlabeled) underlying graph, an integer *x*.
  - *Description:* returns the x-neighbors of the interactors in *S*.
- **xSubGraph()**
  - *Input:* a subset *S* of interactors of the (unlabeled) underlying graph, an integer *x*.
  - *Description:* returns the subgraph containing interactors in *S* and their x-neighbors.
- **xWeightedNeighbors()**
  - *Input:* an interactor *i* of the (labeled) underlying graph, an integer *x*.
  - *Description:* returns the x-weighted-neighborhood of *i*.
- **xWeightedSubgraph()**
  - *Input:* an interactor *i* of the (labeled) underlying graph, an integer *x*.
  - *Description:* returns the subgraph containing *i* and its x-weighted-neighbors.
- **closestComponent()**
  - *Input:* a subset *S* of interactors of the underlying graph.
  - *Description:* returns the interactors forming the connected component of the underlying graph, containing the largest number of elements in common with *S*.
- **intersectionByComponent()**
  - *Input:* a subset *S* of interactors of the underlying graph.
  - *Description:* for each distinct connected component of the underlying graph, returns its unique id number and the size of its intersection with *S*.

IOmanager. The IOmanager class keeps a collection of methods that can be invoked to manage the import/export of MIN from/to external sources. Usually, molecular networks are not stored in the original sources in a format ready to be analyzed by a distributed approach. Instead, they are stored in text files adopting a structured format (e.g., MITAB).

Using the methods available in IOmanager, it is possible to load automatically the MIN from an external file and instantiate it as a distributed data structure.

We consider also another case, less frequent but interesting as well, such that the network is initially stored in a NoSQL database. In particular, we take into account the scenario where nodes and edges are stored in a Neo4j instance. In such a case, it is usually required to properly use the query language to acquire a complete description of the molecular interactions. Again, using the methods available in IOmanager, it is possible to automatically load the MIN from a Neo4J database without the need of writing any query.

The list of methods managing the import procedures is reported in the following:

- importFromTxt()
- importFromNeo4j()

Conversely, it may be required to store the network resulting from an analysis on a device external to the distributed system, allowing to process it by other tools. The library provides the following functions to transfer MIN information on a text file and to transfer or update MIN information on a Neo4j instance:

- exportToTxt()
- exportToNeo4j()
- updateNeo4j()

## Results

In order to assess the performance and effectiveness of the proposed library, different experiments have been performed, as described in the following paragraphs. In particular, a first class of experiments has been devoted to test the efficiency and scalability of DIAMIN. A second one has regarded its ability to solve specific problems by using directly its functionalities (e.g., for data analysis use). Finally, an example use case showing how the DIAMIN functionalities may be used to develop new ones is provided as well.

For the described experiments, two different networks have been considered, referring to Homo sapiens and built upon the interactions retrieved from two databases: Intact [4] and String [5]. Both databases store mainly protein–protein interactions, however they also include a small fraction of interactions between proteins and other molecules (e.g., miRNA). Their main differences is that the former stores interactions experimentally verified only, while the latter includes also interactions computationally predicted.

We denoted by ${{\mathcal {N}}}_I$ the MIN obtained from Intact and $\mathcal{N}_S$ that from String, respectively. ${{\mathcal {N}}}_I$ has 116, 641 nodes and 768, 993 edges, ${{\mathcal {N}}}_S$ has 19, 354 nodes and 11, 759, 454 edges. The tests have been performed on an HPC infrastructure equipped with 8 compute nodes running Linux, each equipped with 2 AMD Epyc 7452 processors and 256 GB RAM, for a total of 512 compute cores (see [18] for more details).

### Performance evaluation

We have assessed the performance of our library, both in terms of efficiency and scalability, by benchmarking it when used to solve a reference use-case. Given a set of interactors $P=\{p_{1},\dots ,p_{n}\}$ of ${{\mathcal {N}}}_S$, performance of DIAMIN has been then evaluated by considering the task of computing, for each $p_{i}\in P$, its **xWeightedNeighborhood**, by fixing $x=0.45$.

Efficiency has been measured by comparing the elapsed execution time of a solution for this task based on our library, against an equivalent solution developed using the Neo4J graph-oriented database (see Background). In this latter case, first we loaded in memory the whole network, then we completed each task by running a properly crafted Neo4J query (a copy of the used queries is included in the DIAMIN documentation). We stress here that, since Neo4J does not support the deployment of fully distributed graphs, the graph under analysis has been loaded and queried using one single server machine.

**Fig. 2:**
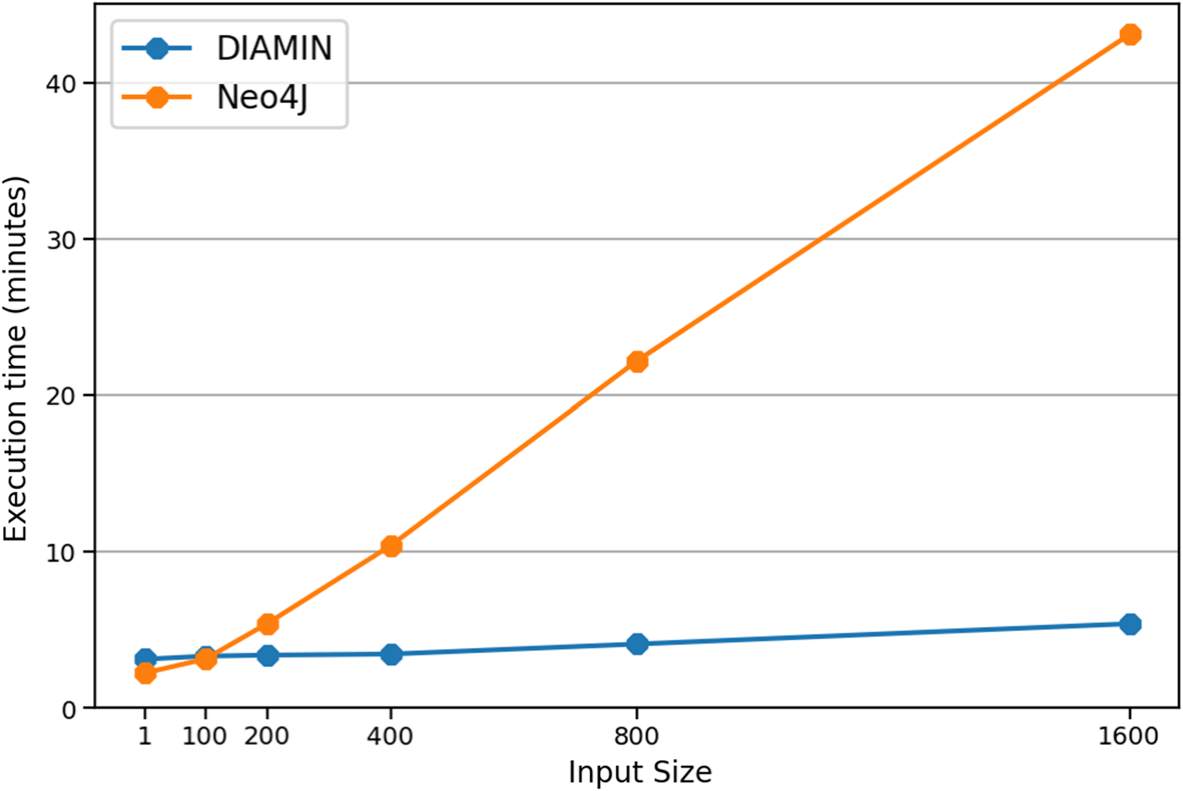
Execution time (in minutes) of our solutions, respectively based on DIAMIN and Neo4J, when executing the considered task, as a function of the number of interactors being analyzed (chosen uniformly at random among those in $\mathcal {N}$)

For both competitors, the considered task has been executed several times, by doubling the size of *P* at each time. Figure 2 shows that the Neo4j solution is strongly influenced by the size of *P*, indeed its execution time increases linearly with *P*, starting when the latter is yet quite small (that is, 100). On the contrary, the execution time of the DIAMIN solution is just slightly influenced by the increase of *P*. Indeed, this behavior can be explained by considering the fully distributed nature of our solution. That is, DIAMIN allows all nodes of the distributed system to explore in parallel the neighborhood of the proteins they hold, including the interactors in *P*. Instead, due to its inherently sequential nature, the Neo4j solution takes a time that is linearly proportional to the number of interactors being considered.

**Fig. 3:**
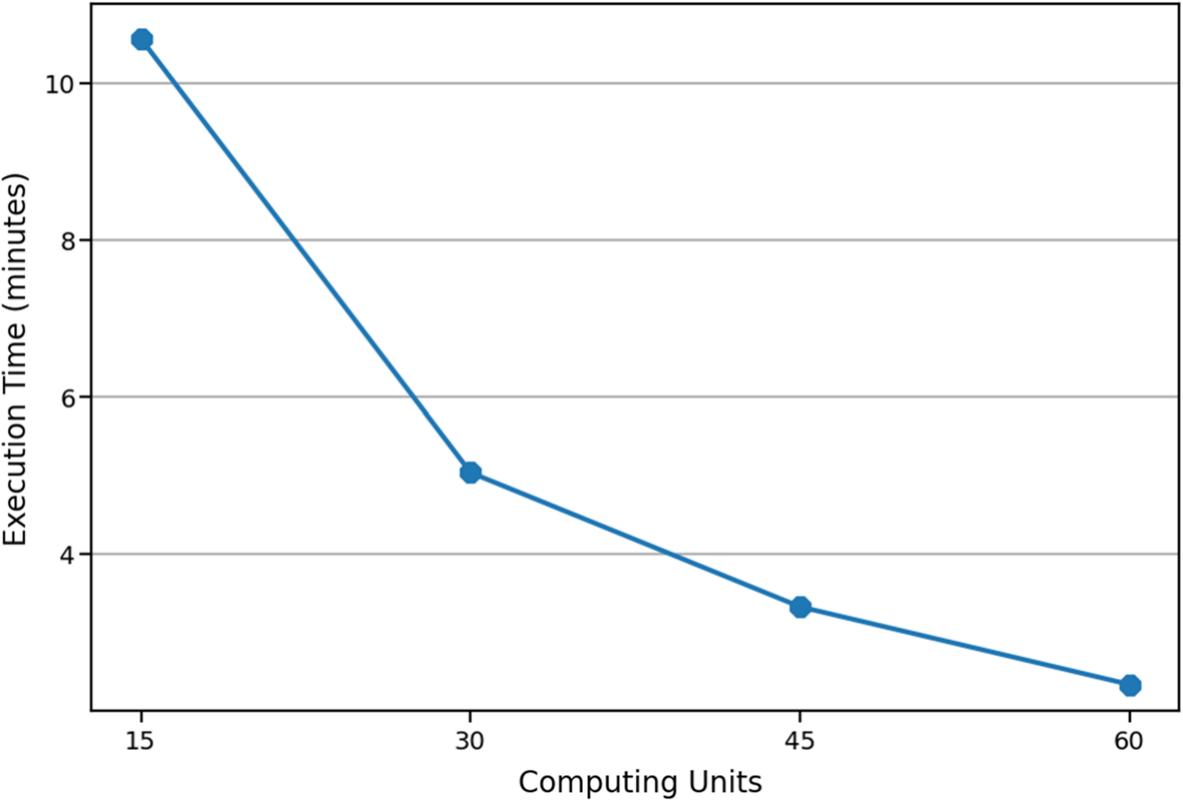
Performance scalability of our DIAMIN-based solution, when accomplishing our reference Task, considering a set of 1, 600 interactors chosen uniformly at random from $\mathcal {N}$ and using an increasing number of computing cores

Scalability has been measured by analyzing the elapsed execution time of our solution for this task, as a function of the number of computing cores. As shown in Fig. 3, our distributed solution succeeds in achieving a nearly linear scalability when accomplishing this task, thanks to its ability to process large graphs in a truly distributed way.

### Direct use of existing functions

Here two case studies are discussed regarding the solution of specific data analysis problems, by using directly the primitives included in DIAMIN.

#### Example 1

The top hubs in the network The search of the most central nodes/edges in biological networks has received wide attention in the literature (e.g., [19–22]). In a MIN, pivotal interactors are likely to be represented by highly connected nodes (i.e., hubs). The degrees() function of our library allows the user to extract a subset of interactors, according to the value of their degree. This function computes the degrees of each interactor and it returns all those elements satisfying a given condition. Listing 1 shows how to return the interactors associated with the 20 largest degrees (in case of ties, the nodes exceeding the required amount, are left out).


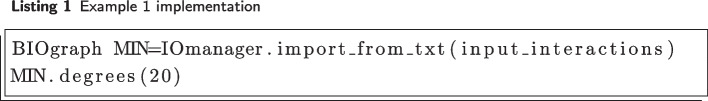


**Table 1:** Interactors for *Homo sapiens* associated with the 20 largest degrees, respectively in the Intact and in the String databases

	Intact		String	
	Node ID	Degree	Node ID	Degree
1	uniprotkb:P42858	6807	uniprotkb:P04406	15,014
2	**uniprotkb:Q5S007**	4512	uniprotkb:P60709	13,880
3	uniprotkb:P13569	4291	uniprotkb:P31749	11,836
4	uniprotkb:P03372	3574	**uniprotkb:Q5S007**	11,608
5	uniprotkb:P54253	2231	**uniprotkb:P04637**	11,304
6	**uniprotkb:P05412**	2188	uniprotkb:P01106	11,226
7	uniprotkb:P19320	2060	uniprotkb:P35222	10,142
8	uniprotkb:Q08379	2027	uniprotkb:P02768	9990
9	uniprotkb:P62993	1736	uniprotkb:P00533	9024
10	uniprotkb:A8MQ03	1663	uniprotkb:P07900	8872
11	uniprotkb:Q6FHY5	1651	uniprotkb:P01112	8824
12	uniprotkb:P60410	1578	uniprotkb:P27361	8600
13	uniprotkb:Q99750	1531	uniprotkb:P12931	8418
14	uniprotkb:P00533	1506	uniprotkb:P01308	8290
15	**uniprotkb:P04637**	1493	uniprotkb:P01116	8234
16	uniprotkb:O76024	1484	uniprotkb:P60484	8214
17	uniprotkb:Q7Z699	1464	uniprotkb:P34932	8170
18	uniprotkb:Q8TBB1	1456	**uniprotkb:P05412**	7874
19	uniprotkb:P05067	1451	uniprotkb:P01375	7774
20	intact:EBI-25847655	1437	uniprotkb:O15550	7562

Interactors appearing in both rankings are drawn in bold

Table 1 shows interactors associated with the 20 largest degrees in ${{\mathcal {N}}}_I$ and ${{\mathcal {N}}}_S$, respectively. A first observation is that, as expected, the values of node degrees are much larger in ${{\mathcal {N}}}_S$ than in ${{\mathcal {N}}}_I$, due to the fact that String contains a largest number of interactions than Intact, including also those predicted computationally. Moreover, ${{\mathcal {N}}}_I$ and ${{\mathcal {N}}}_S$ share three of the nodes in the top-20 degree classification. In particular, such nodes are:

- LRRK2 (uniprotkb:Q5S007), that is, a leucine-rich repeat serine/threonine-protein kinase 2, involved in multiple processes such as neuronal plasticity, autophagy, and vesicle trafficking (see, e.g., [23]).
- JUN (uniprotkb:P05412), a transcription factor AP-1 that recognizes and binds to the enhancer heptamer motif 5’-TGA[CG]TCA-3’ [24].
- TP53 (uniprotkb:P04637), that is, cellular tumor antigen p53 which acts as a tumor suppressor in many tumor types, and induces growth arrest or apoptosis depending on the physiological circumstances and cell type [25].

#### Example 2

*Neighborhood functional analysis* The analysis of neighborhoods of specific nodes in a MIN is a very common task in the literature (e.g., [26–29]). The function with name xWeightedNeighbors() returns the x-weighted-Neighborhood of an input node. Listing 2 shows the instructions to perform this task. Table 2 shows the neighborhoods obtained for the protein *TP*53 (uniprotkb:P04637) at different values of *x* (0.70, 0.75, 0.80 and 0.85, respectively), with reference to the reliability scores returned by Intact for each interaction.


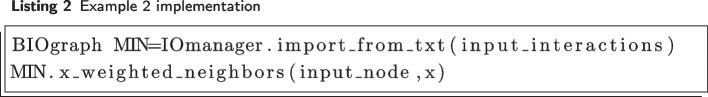

**Table 2:** Neighborhoods obtained for the protein *TP*53 (uniprotkb:P04637) at different values of *x*, w.r.t. the Intact reliability scores

x	Neighborhood
0.70	AKT1, APEX1,ARRB2,ATRX,BAD,BAK1,BAX,BBC3,BCL2L11,BHLHE40,BID,
	BRD7,BRI1,CCAR2,CCND1,CDK2,CDKN2A,COP1,CREBBP,CSNK2A1,CUL7,
	CUL9,Crebbp,DAPK1,DAXX,DLG1,DNMT1,DVL2,E6,EBNA1,EEF1A1,EGFR,
	EP300,FYN,GTF2H1,H4-16,H4C1,H4C11,H4C12,H4C13,H4C14,H4C15,H4C2,
	H4C3,H4C4,H4C5,H4C6,H4C8,H4C9,HDAC1,HGS,HIF1A,HIPK2,HMGB1,
	HNRNPUL1,HSPA9,HTRA1,HTRA4,HTT,ICP0,IKBKB,IKBKG,IRF3,L,LCK,
	LRRK2,Lrrk2,MAGEA2,MAGEA2B,MAP3K5,MDM2,MDM4,MKRN3,MYH9,
	NCL,NME1,PARP1,PBK,PCNA,PIN1,PLK1,PML,POLA1,PPP1CA,PPP1CC,
	PPP1R13L,PSMD4,PTK2,Ptk2,RAD23A,RANGAP1,RB1,RBCK1,RCHY1,
	RELA,RIPK3,RNF31,RPL11,RPS19BP1,RPS7,S100A4,S100B,SERK1,SETD7,
	SHARPIN,SIRT1,SMYD2,SQSTM1,STAM,SUMO1,Sirt1,TBP,TFB1,TOP1,
	TP53BP1,TP53BP2,TPT1,TRAF6,TWIST1,Tp53,UBC,UBE2D2,UBE2I,
	UBE3A,USP7,VRK1,WRN,Wasl,abrB,cph1,degP,kaiC,kinA
0.75	AKT1,BAK1,BAX,BBC3,BCL2L11,BID,CDKN2A,CREBBP,Crebbp,DAPK1,
	DAXX,DVL2,E6,EBNA1,EGFR,EP300,HGS,HIF1A,HSPA9,HTRA1,IKBKB,
	IKBKG,IRF3,LRRK2,MAP3K5,MDM2,MDM4,MKRN3,MYH9,NME1,PARP1,
	PCNA,PIN1,PPP1CA,PPP1CC,PPP1R13L,PSMD4,PTK2,RAD23A,RB1,RCHY1,
	RPL11,S100A4,SETD7,SIRT1,SMYD2,SQSTM1,Sirt1,TBP,TP53BP1,TP53BP2,
	TWIST1,Tp53,UBC,UBE2D2,UBE3A,USP7,VRK1,cph1,degP,kaiC,kinA
0.80	AKT1,BAK1,BAX,BCL2L11,CDKN2A,CREBBP,Crebbp,DAXX,E6,EGFR,EP300,
	HGS,HIF1A,HSPA9,IKBKB,IKBKG,IRF3,LRRK2,MDM2,MDM4,NME1,PIN1,
	PPP1R13L,PSMD4,RAD23A,RB1,RCHY1,RPL11,S100A4,SETD7,SIRT1,SMYD2,
	SQSTM1,TP53BP1,TP53BP2,TWIST1,Tp53,UBC,UBE2D2,USP7,cph1,degP
0.85	BAX,CREBBP,Crebbp,DAXX,EGFR,EP300,HIF1A,IKBKB,IRF3,LRRK2,MDM2,
	MDM4,NME1,PIN1,RB1,RCHY1,RPL11,S100A4,SETD7,SIRT1,TP53BP2,Tp53,
	UBC,USP7,cph1,degP

We now show how, for example, it is possible to exploit the obtained neighborhoods in order to perform Functional Enrichment Analysis. It is worth pointing out that this type of analysis is particularly interesting in the case of weighted neighborhood, due to the fact that the nodes involved in the neighborhood are not simply at a “fixed distance” from *TP*53 in the network (e.g., by paths of a fixed length). Instead, they all share the property to be linked to *TP*53 by paths which preserve a given degree of reliability, guaranteed by the fact that the product of labels of the corresponding edges cannot exceed the value *x*.

Functional Enrichment Analysis has been performed on the largest neighborhood shown in Table 2, by using the enrichment analysis service, available on the Gene Ontology (GO) [30] website, using the analysis tool from the PANTHER Classification System [31]. Gene sets with a *p*-value less than 0.05 have been considered significantly enriched, with references to each of the three GO vocabularies, i.e., biological process, molecular function and cellular component. In particular, genes coding for the proteins in the considered set are significantly involved in several biological processes, such as B cell negative selection, apoptotic process involved in embryonic digit morphogenesis, positive regulation of macrophage apoptotic process and others. Moreover, they have also different molecular functions in common, e.g., enzyme binding, kinase binding and nitric-oxide synthase regulator activity. On the cellular component vocabulary, the enrichment analysis has returned mitochondrial outer membrane, PML body, cytosol and others.

### Implementation of new functions

DIAMIN also allows user-driven analysis of MIN. Indeed, the provided functions can be suitably combined, or usefully modified, in order to implement new algorithms that solve both standard and more specific problems in network analysis. As an example, many algorithms proposed for network alignment are based on neighborhoods exploration (e.g., [26–29]), therefore DIAMIN functions for neighbors and closeness computation may be useful to carry out network alignment on very large MINs using a distributed approach. On the other hand, many algorithms have been proposed in the last few years for biological networks clustering (e.g., [11, 32–35]). Again, DIAMIN functions for subgraphs and components extraction may be useful to implement novel algorithms for very large MINs clustering.

Another interesting problem in biological network analysis is the study of node/edge centrality in the network, that is both interesting per se and considered in some cases as a sub-task for the solution of other problems (network alignment and clustering, for example).

In Example 1 we have discussed a very basic type of node centrality, that is, node degree. However, also more complex centrality measures have been introduced in the literature [36]. In Example 3 we show how it is possible to use DIAMIN for computing one of the latters efficiently.

#### Example 3

Kleinberg dispersion computation Given a MIN ${{\mathcal {N}}}=\langle V, E \rangle$, a pair of nodes $u,v \in V$ and their corresponding sets of neighbors $C_{u}$ and $C_{v}$, the Kleinberg dispersion [37] between *u* and *v* is defined as:

$$\begin{aligned} K(u,v)=\sum _{s,t \in C_{uv}}d_{v}(s,t) \end{aligned}$$

where $C_{uv}$ is the set of common neighbors of u and v, *C* is the cardinality of $C_{uv}$ and $d_{v}(s,t)$ is the function equal to 1 if *s* and *t* belong to different connected components of $C_{u}\setminus \{u, v\}$, and equal to 0 otherwise. In particular, the Kleinberg dispersion takes into account both the size and the *connectivity* of *u*,*v*’s common neighborhood. Intuitively, it quantifies how *“not well”*-connected is the *u*,*v*’s common neighborhood within $G_{u}$, i.e., the subgraph induced by *u* and its neighbors.

**Fig. 4:**
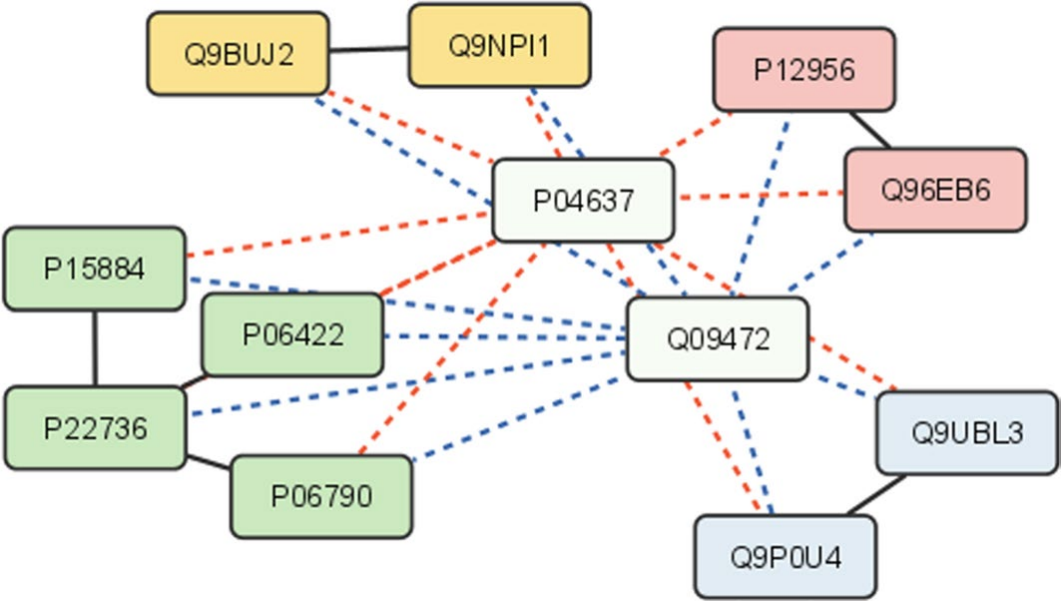
Subnetwork extracted from ${{\mathcal {N}}}_I$. The proteins *uniprotkb:P04637* and *uniprotkb:Q09472* represent bridge-like nodes between the four components denoted with different colours and *K*(*P*04637, *Q*09472) is equal to 56. Conversely, *K*(*P*04637, *P*06422) is equal to 0 since their common neighbors (*uniprotkb:P15884*,*uniprotkb:P22736*,*uniprotkb:P06790*) belong to the same component

By exploiting the DIAMIN functions, *K*(*u*, *v*) can be easily computed with the short pipeline shown in Listing 3. Figure 4 illustrates the Kleinberg dispersion computation on a small graph.
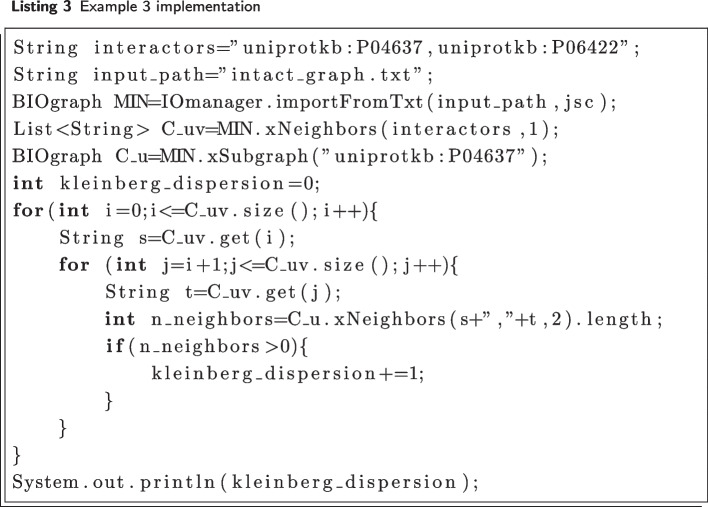

## Conclusion

We have presented a high-level software library designed and developed to allow users without any distributed programming skill to perform data analysis, and expert programmers to implement new algorithms, on large-scale molecular interaction networks. The library has been tested on data retrieved from the Intact and String databases, showing to be highly efficient and scalable. Moreover, we have provided different examples for its usage.

In the future, we plan to approach specific problems for the analysis of molecular interaction networks by implementing novel algorithms in the distributed based on the primitives provided by DIAMIN. In particular, an interesting problem to deal with is multiple network alignment, which is well suited to be faced in a distributed environment.

Finally, we plan to extend this project, hopefully including further functionalities designed and developed also by other researchers working on molecular interaction networks.

## Data Availability

The library runs over Apache Spark 2.4 (or higher version), and requires a Java compliant virtual machine 1.8 (or higher version). A copy of DIAMIN is freely on a repository reachable at https://github.com/ldirocco/DIAMIN. It contains: The source code of DIAMIN. The links to the datasets used for testing DIAMIN. A README file, containing all information needed to set up and use our library, including: (i) explanation on how to run the examples mentioned in the manuscript, and (ii) instructions for using the library by command line. A manual where the main provided functions are described in detail, together with useful examples on how to run them in local or on the cloud. A collection of web pages, generated according to the JavaDoc format, providing a quick reference to the DIAMIN API.

## Abbreviations

MIN: Molecular interaction networks
PSU: Users with programming skills
DAU: Data analysts users
RDD: Resilient distributed datasets
